# Copland, Kennedy, Lorimer, Holland, on the Causes and Propagation of Asiatic Cholera

**Published:** 1847-04

**Authors:** 


BRITISH AND FOREIGN

### MEDICAL REVIEW,

FOR APRIL, 1847.

### PART FIRST.

### Analytical and Critical Reviews.

### Art. I.

1. *A Dictionary of Practical Medicine.* By James Copland, m.d., f.r.s. Part X. Article "*Pestilence Choleric.*" —*London,* 1846.
2. *Notes on the Epidemic Cholera.* By R. H. Kennedy, m.d., late Physician-General and President of the Medical Board, Bombay. Second edition.—*London,* 1846.
3. *Report on the Epidemic Cholera, as it has appeared amongst the Native Troops of the Madras Army on the Line of March from one Station to another, from* 1820 *to* 1844 *inclusive.* Drawn up by Alexander Lorimer, m.d. Published by Order of Government.—*Madras,* 1846.
4. *Medical Notes and Reflections, &c.* By Henry Holland, m.d., f.r.s. Chapters, "*On the Method of Inquiry as to Contagion*" (pp. 276), and "*On the Hypothesis of Insect Life as a Cause of Disease*" (pp. 567). Second edition.—*London,* 1840.

The investigation of the rise, progress, and nature of the disease commonly called "Asiatic Cholera," is one of the great medical problems of our time. Our interest in the subject is testified by the variety of works which have been published upon it, and by the labour which has been spent in examining it. And to those who are acquainted with the literature of cholera it will appear that a considerable amount of real knowledge has been attained. The observation of nearly thirty years has given us a mass of evidence in a great measure yet unanalysed, from which future inquirers may venture to make a safe and tolerably complete generalization. Still it cannot be denied that at present great uncertainty and obscurity exist, with regard to many of the most important points, and that the opinions of men of eminence are widely at variance. In some instances, this is probably inevitable from the difficulty of the subject, but in other cases there can be little doubt that erroneous conclusions have been arrived at, from a want of due care in the examination of evidence and of recorded observation.

Between the history of cholera and that of previous epidemics there exists a wide and singular difference. Never before have we had an opportunity of obtaining an authentic record of the very commencement and origin of a pestilence. The ravages of the "Black Death" and of the "Sweating Sickness" occurred in a comparatively unenlightened age, and first made their appearance among rude and semi-barbarous nations. Their origin was concealed by the clouds of geographical ignorance, their nature defied the inquiry of an unformed physiology, and an imperfect chemistry. From the scattered notices and records of contemporary writers modern industry has compiled only an imperfect and disjointed narrative of their progress.

The history of cholera as an epidemic is, on the contrary, the completest in the records of medicine. Two hundred years ago, and it would have swept over the earth and again have disappeared. uninvestigated and misunderstood. But the remarkable position of the English in India, has afforded the opportunity of observing the apparent commencement of this disease in 1817, in the hot and humid atmosphere of Bengal, and of tracing with great accuracy each step of its variable and capricious progress.

It might have been anticipated that this minute investigation would have thrown no little light on the obscure subject of epidemic diseases generally. We might have imagined that some clue would be given us as to the mode in which the great subject of the spread of contagious and infectious diseases should be studied. But, on the contrary, it has increased the difficulty of the subject; another problem is given to the inquiry; another complex affection is added to the list of those diseases, dissimilar in their origin, their nature, and their treatment, but which are connected together by the one single tie of universality of prevalence.

The whole subject of epidemic diseases is, indeed, one of peculiar difficulty. The subtle agents active in their production, are for the most part unrecognized by science. The various conditions in which the bodies receiving these agents are placed, either by changes in themselves, or by physical alterations in surrounding media, are undetermined or but vaguely guessed at. The division which has been made of them into contagious and non-contagious has simplified the subject only in appearance. In reality it has left untouched the real difficulties of the case. It may be that hereafter it will be found to be little more than a verbal distinction, which nothing but ignorance of the subject could have introduced.

Before entering on the subject of the contagion of cholera, it may be as well to devote a few pages to the general consideration of contagious and epidemic agents.

The use we are about to make of that chapter in Dr. Holland's philosophical work which is entitled the 'Method of Inquiry as to Contagion,' has induced us to place the name of his book at the head of our article. We have no intention, however, to enter into anything like a review of this able work; this would be foreign to our present purpose, even if we had not previously noticed it. Suffice it to say that by its philosophical tone, and by its acute and refined criticism, it is a work destined evidently to exert a durable influence on English medical literature.

A remark made by Dr. Holland seems to us the solution of the uncertainty and variety of opinion which exists on the subject of contagion.

"In all common reasoning on the subject, and even in what has been written upon it, infection is too much regarded as a simple and uniform act; and the virus transmitted as the same in quantity and intensity. Such views, however, carry error into every part of the discussion. (Holland, p. 280.)

The full meaning of this passage can only be elucidated by a short analysis of the difficulties and obscurities surrounding the inquiry into the laws of contagion.

The most common notion of diseases contagious, and diseases epidemic but not contagious, we take to be the following. In both cases a *virus* is assumed, a *materies morbi* of undetermined nature which propagates itself when it meets with its conditions of development. In the simply contagious disease, this virus or morbid agent does not lose its power of propagation by transmission through the animal economy; it continues even to propagate itself still more, and to increase, if not in intensity, certainly in amount. In the other case, the agent exhausts itself on the individual; the changes it leads to, annihilate itself; another stock of virus from the original source is requisite for the attack of another person. In both cases, at certain times, each agent acquires an extraordinary power of development. The conditions producing this activity are but partly known, and have generally been referred to unusual deviations in the composition or ordinary states of the atmosphere.

The first question which occurs respecting this opinion is the assumption of a materies morbi, or poison. In the case of contagions communicable by inoculation we admit at once the existence of a virus; in the case of contagions which clearly infect the majority of persons, in the vicinity of diseased people, we also admit the virus, although it is incommunicable by inoculation; in the case of a disease, endemic and local, such as ague, we also analogically admit the virus, and consider it in this case existent in the atmosphere. But in other cases of diseases undoubtedly epidemic, the presence of the materies morbi is more doubtful. For example, at certain times and places, dysentery becomes so general as to be entitled to the appellation of epidemic. It affects people whose habits and modes of living have not in any way conduced to the disease, and it is referred, therefore, to some peculiarity in the atmospheric conditions; to great humidity; to sudden and unequal vicissitudes of temperature; or to the prevalence of certain winds, and other changes of a similar kind. This explanation is probably correct, and if so, it affords us an example of a disease spreading over large districts of country, on account of changes produced in the animal system, by causes acting, so far as we can understand, upon the secretions and processes of assimilation and nutrition. Here the atmosphere has produced an epidemic, and it is not possible to draw a distinction between this and another arising from less obvious causes and spreading over a wider extent of country, because this would be to assume that we are acquainted with all the alterations in the atmosphere, and can accurately measure its variations. The question stands thus: we see that certain atmospheric changes, for the most part obvious enough, are sufficient without reference to a supposed virus to induce in a great number of people a peculiar disease ; how is it to be proved, that analogous changes which may be to us more obscure and latent, do not produce other affections, by means of similar obscure influences on the processes of nutrition ?

In this position now remains the subject of cholera. Some would refer it entirely to atmospheric changes ; others to a material poison requiring for its propagation certain conditions of the atmosphere, but not reproduced by the human body ; and a third class of observers would make it a poison reproducing itself by transmission like smallpox, and merely influenced as this disease is, by atmospheric states favorable to its generation.

At the outset of the inquiry respecting diseases decidedly contagious, difficulties arise which indicate at once how scanty is our real knowledge, and how inefficient are our means of extending it. The question we have proposed is the determination of the presence or absence of certain specific agents ; difficult as this is, it becomes much more complicated, if we allow that the two classes of agents are convertible into one another. Thus, a certain class of reasoners, endeavouring to account for the various phenomena manifested in cholera, in continued fever, and other diseases, have imagined that a disease, at first non-contagious and of atmospheric origin, may gradually acquire the property of being transmitted from body to body, or may give rise to an agent capable of doing it ; that at different periods of the disease, this primary or secondary agent may manifest different and apparently irreconcilable properties, being at one time expended in the system, and at another, deriving from this system increase of power; at one time annihilated, at another generated, and under both conditions producing an identical pathological state.

It is obvious, that if possible, this question should first be settled ; otherwise endless fallacies may be introduced into the inquiry. But it is questionable whether it is at present susceptible of determination : arguments and suppositions on either side balance each other, and the opinions of those well informed on the subject, are in all probability equally divided.

The instance of continued fever completely illustrates the case of cholera considered in this light. Everybody admits a continued fever, arising from local circumstances, from vegetable effluvia, from malaria,—from, in fact, a modification of the causes of remittent fever ; occasionally, also, a continued fever seems to arise simply from atmospheric vicissitudes. Neither of these can be considered as at first contagious ; yet it is the general opinion, and one founded on long experience, that a fever exists in this country, which is undistinguishable, as far as symptoms and morbid appearances are concerned, from the two former affections, and which yet is contagious. Is this fever distinct from the former, or is it a product of them ? We think that no one can doubt, after an examination of the evidence on this point, that a contagious property may be attached at a certain stage to a fever, which at first was not merely evidently caused by malaria, but which presented even the characters of a true remittent. There is equal evidence to prove that a fever apparently similar may be contagious from the commencement. Here we see two causes, seemingly different, producing apparently an identical disease: although all reasoning on the subject would hold this to be almost impossible.

Some would here make a distinction between contagions, and would assume that the agents of smallpox, measles, or scarlatina are to be distinguished from the more undefined contagion of typhus, as being strictly generated from similar agents which pre-exist, and have never been proved to arise *de novo.* And they remark that these contagions possess also the remarkable power of destroying in the system the property it at first possessed to be affected by, and to reproduce, them.

Great as this difference is, it may be questioned whether much influence ought to be attached to it. Among the diseases themselves which are thus separated, we find differences as striking as those supposed to distinguish them from the comparatively irregular and recurrent typhus. The same virus produces apparently the mere local affection of the fauces and the true or regular scarlatina; yet one disease is recurrent and the other not; smallpox transmitted through the system of a brute undergoes the remarkable change of being afterwards able to be propagated only by actual contact. Between the different poisons of smallpox, scarlatina, and measles are found also great differences in point of diffusion, adherence to clothes, &c. It may be questioned also whether this non-recurrence of a disease is ever quite complete; we know it not to be the case in cowpox, which gives immunity only in certain cases, or, perhaps, for a certain time; perhaps smallpox would, after a longer interval than human life allows it, also return. It is not proved, on the other hand, that typhus does not give a shorter period of exemption; some writers hold this opinion, which if correct, would at once remove this distinction, as far as recurrence is concerned, between itself and the contagious exanthemata. Sir W. Pym and others are strong advocates for this opinion in the case of the "contagious" yellow fever.

The points we have alluded to give us some insight into the difficulty of the subject;—we see great changes occurring in the poison of smallpox by the action of the living system of the cow. If our experience were greater, other diseases of brutes might become known to us, as derived from diseases in man, analogous but not identical. Rabies in the dog again produces in the human body a disease distinctly modified by the transmission. If the chemistry of vitality possesses this remarkable power, why should we doubt that out of a non-contagious miasma it may generate another principle possessing the power of transmission?

At any rate, considering our ignorance, it would not be right to deny the possibility of this occurrence; and yet, admitting it, it seems hopeless to attempt to comprehend principles which, for anything we know, may be continually varying in Nature, although their appreciable effects remain the same.

Leaving this question of "convertibility," we find another difficulty almost as great, which it is necessary fully to investigate before much progress can be made in the inquiry. We have hitherto alluded only to the virus itself, but great modifications are produced in each contagious agent by the surrounding media. It is generally admitted that the effluvia from persons affected with smallpox, measles, &c., being diffused through the atmosphere become innocuous when diluted to a certain extent. This extent is not accurately known, but appears to differ considerably in the different well-known instances of contagion. We have a right to assume that this extent will not merely depend upon the virulence, intensity, or abundance of the emanating virus, but that it will also depend upon the properties at the time of the medium with which it is commixed. That is to say, we see during certain years that, apparently from unexplained and occult conditions of the atmosphere, the virus of smallpox acquires an unusual power of development; that which happens thus to the whole body of the poison, may continually occur in those detached portions of it, which emanate from an infected person. We can conceive, then, either a rapid or a slow diffusion of a contagion, and it is not too much to assume that, in the case of smallpox, if by any chance those conditions of the atmosphere which favour its diffusion were to be absent, it might, for a time, become a disease propagated only by contact and inoculation. On the other hand, it does not require much stretch of imagination to conceive that the presence of some of its conditions of existencc in unusual abundance, might cause a virus to be so rapidly diffused and dissipated as to be incapable of inoculation, and to become only infectious.

It has been observed that under certain circumstances smallpox cannot be inoculated. Dr. Copland noticed this on the west coast of Africa. The same circumstance is recorded by Norris, in the 'Philosophical Transactions for 1781,' (p. 53.) He states that, during the prevalence of the Harmattan on the African coast, smallpox is not contagious.* It has also been observed that the virus of cowpox loses its activity in particular climates in India. And this is not owing to modifications produced by heat in the dried virus, because it is propagated with difficulty even when one case has accidentally succeeded; and, in addition, virus similar to that which proves unsuccessful during one season will succeed in another. Dr. Copland states, on the other hand, that in the hot, moist, and sultry weather, following the rainy season, on the African coast, the variolous poison acquires an extraordinary power of development, and is extensively diffused and fatal.

Once admit that atmospheric conditions will modify a virus to this extent, and the whole question of contagion becomes expanded and altered. Pushed to its legitimate conclusion, this view would consider contagion as simply an accidental property impressed upon a poison by conditions which are necessarily variable and uncertain.

Without going to this length we may feel justified in doubting how far

* Dr. Dobson, in whose paper Mr. Norris's observations are recorded, remarks, "that in the year 1770 there were on board the Unity, at Uhydah, more than 300 slaves; the smallpox broke out among them, and it was determined to inoculate. Those who were inoculated before the Harmattan come on got through very well. About 70 were inoculated a day or two after the Harmattan set in, but not one had either sickness or eruption. It was imagined that the infection was effectually dispersed and the ship clear of the disorder, but in a very few weeks it began to appear among these 70. About 50 were inoculated the second time; the others had the disease in the natural way." (Philosophical Transactions for 1781, p. 54.) The Harmattan has also, according to Mr. Norris, a beneficial effect on all disorders. It is an extremely dry, but not a hot wind. The thermometer during its prevalence seldom averages more than 80 degrees at midday. On account of its dryness vegetables are much injured by it.

the division into diseases communicable only by contact, and diseases transmissible without direct contact and inoculation, may not be hereafter proved to be only an expression of greater or less volatility and diffusion.

If the phrase "conditions of existence," which we have more than once employed, be a correct one, it implies several important laws of contagion. It implies that a virus from want of such conditions may be destroyed or rendered inert, as has been said to be the case with typhus and contagious yellow fever, which are destroyed by very hot or by cold weather respectively. It implies also, not mere existence, but development and increase of a virus, and this consideration leads to the most important, because the most practical, part of the inquiry into contagion; it leads to the question whether a contagious virus is capable of indefinite multiplication, apart from well-known multiplying sources like the animal system. If this is admitted, it becomes almost impossible to make use of the usual evidence which has generally been considered sufficient to determine the question of the contagion of a particular disease.

"It is a frequent mistake," writes Dr. Holland, "in reasoning upon contagion to consider that the infectious nature of a disease may be disproved by showing that it has been spread without any obvious communication through man or human means. The two conditions brought into the question are, in fact, perfectly compatible with each other. If a virus can be transmitted from the body through a few feet of air, we are not entitled, from the partial experiments hitherto made, to set any limit to the extent to which, under favorable circumstances, it may be conveyed through the same or other medium. Common reason here concurs with our actual experience of the transmission of the virus of certain diseases in various ways and to remote distances." (Holland, p. 281.)

If this view be correct, and we do not feel disposed to contest it, much greater scope must be given to the idea of contagion. The fact of a person falling sick who could not possibly have been exposed to the exciting cause of a disease, has hitherto been considered strong negative evidence that the disease in question was not propagated by the human system, but was of atmospheric or other non-contagious origin. But such evidence under this view is worth nothing, and, in fact, it becomes difficult to disprove the presumed contagious nature of any disease spreading epidemically.

If it be allowed that a germ of contagious matter thrown off from the body of a diseased person may be carried to an indefinite distance, and may still possess (or *acquire?*) sufficient intensity to affect individuals who have not been within the usually recognized sphere of infection, then it must be admitted that quarantines, cordons, and similar attempts to localize the contagious virus, are founded on a misconception of the laws of infection; and that the only true way of preventing the spread of a disease is by recognizing, and as a consequence, removing its conditions of development.

We have entered thus far into this very obscure and extensive subject, with a view of indicating some of the difficulties which appear to us to stand in the way of its full investigation. But we are conscious that many eminent and philosophical inquirers will consider that we have imputed to the subject a vaguer and more indefinite character than really belongs to it. To take as an example two writers on cholera, whose names appear at the head of this article, Drs. Copland and Kennedy: both these gentlemen are firm believers in the contagious nature of cholera, and in the most limited sense of the word. Contagion, according to them, implies either contact or immediate proximity, and the virus requires to act on living systems, to insure its increase and activity. Those states of the atmosphere produced by changes in itself, or by emanations into it from the soil, or from animal or vegetable decomposition, and which we have termed conditions of existence, are considered to have only a limited effect, and as not essential to the development and action of the materies morbi. When the disease spreads they trace intercourse in every case, and Dr. Copland explicitly denies that cholera has ever entered a town which had not held intercourse with an infected place.

"Those who argue against its transmissible nature cannot show a single instance of its appearance in any place without the previous communication with an infected place or persons of a nature to propagate the malady." (Copland, op. cit. par. 93.)

This view of contagion has, at any rate, the merit of being more defined and precise than the one we have already discussed. Attempts have been made to simplify it still more. Many writers are inclined to deny the power of conversion which we have attributed to the living system. They argue that all contagions are fixed, definite, and inconvertible, and once arising *de novo*, probably from animal sources, ever after remain gradually increasing or decreasing according to circumstances.

If this could be proved, the question would at once assume a more decided form and a narrower basis. It appears to us that Dr. Copland has adopted this view, and has laboured to support it with great consistency in all his writings on contagious diseases. In the same category with smallpox, measles, &c., he would include yellow fever, cholera, plague, and typhus. Thus in the case of yellow fever, although he is obliged to admit that a malignant remittent fever will arise from malaria, he yet endeavours to distinguish this from true yellow fever, which he supposes to spring from a contagion of its own, to be a disease *sui generis*, and not capable of recurrence. He distinguishes the "typhoid fever" from true typhus, the one arising from malaria, the other from a specific contagion, and does not allow that one is convertible into the other. So also with cholera; he endeavours to prove its true contagious nature, and is evidently strongly inclined to believe that it only occurs once during life.

Although we cannot regard the definition of contagion as at all settled, and believe that great differences of opinion exist in the minds of many observers as to the full meaning of the word, it is obviously necessary that in entering into the evidence of contagion, as far as cholera is concerned, we should have some decided understanding on the point in question. We will therefore adopt, for the time, the limited and restricted definition of contagion, as implying either contact or proximity. This is the sense in which it is received by the two authors who have most recently written on the subject. It is a view of the question which they profess to prove by evidence, and presents therefore a tangible and defined point for discussion.

In his present article, Dr. Copland reproduces the evidence which he brought forward in 1832, to prove the contagion of cholera. He is also entirely consistent with the view he has taken of contagion generally; he allows no compromise, and makes no reservation: cholera spreads by human intercourse alone, and derives its existence or action from the living body only.

Dr. Kennedy's work is a reprint of one published at Calcutta in 1826. His arguments for contagion are entirely drawn from the Bombay Medical Report, published in 1819. Although an ardent contagionist, he does not seem to have been able to collect any personal evidence of the fact; and the experience of twenty years, since the first edition of his work, has apparently suggested to him no new argument in its favour.

It is admitted by all parties that cholera is not communicable by inoculation. The observations on this point are perfectly conclusive, and indeed undisputed. It is necessary to say a few words on the precautions necessary in examining into the evidence of a disease which is contagious, but not communicable by inoculation.

We have alluded to the difficulty incident to the subject from our ignorance of the real nature of these presumed morbid poisons, of their alterations, modes of development, and conditions of existence. But in tracing their effects on the system, we find no less obscurity and difficulty produced by our ignorance of the state of the system necessary for their manifestation. If we were doubtful about the agent, we are still more doubtful about the recipient. Of one thing we seem to be certain; that there must be a peculiar condition of the body, a predisposition before the agent can act; and this predisposition, in the case of the better known contagions, is all but universal; every person has it, and yet immediately after it has developed the disease, it is lost, obliterated, and future exposure to the virus produces no return of it. What this predisposition is, in the case of smallpox, measles, &c., we are totally ignorant; the recondite processes of sanguifaction and nutrition concerned in its production and destruction are unrecognized by our present science.

Assuming cholera to be contagious, a predisposition of body must be also assumed. It is also fair to believe that this predisposition is often absent. We know that in the case of smallpox it is sometimes absent. It is impossible to define or to limit the frequency of its absence in the case of cholera.

Admitting this, we must also admit that in examining the evidence of contagion we must anticipate a great deal of negative evidence; we must expect many instances of immunity in persons exposed to the virus, but who wanted the predisposition.

We proceed, then, briefly to consider the evidence of the contagion of cholera, using the term conventionally in its restricted sense, as implying either contact or proximity.

The principal evidence on which a disease incommunicable by inoculation, is yet supposed to be contagious, is chiefly derived from the following sources:

1. It is found that those most exposed to the risk of infection, viz. medical men, friends of the sick, and hospital attendants, show a greater percentage of attacks than persons not so exposed. It does not follow that this increased percentage should be considerable, but it must be uniform and constant.

2. Individuals or bodies of men infected with the disease are capable of carrying it from place to place, and of communicating it to the fixed inhabitants. Fixed inhabitants never receive this disease unless it has been imported by the arrival of infected individuals. It is admitted, however, that it is always a difficult matter to trace the spread of the disease from such infected persons.

3. Individuals or bodies of men preserving a strict quarantine, must necessarily show a diminished percentage of attacks. Allowing that incidental and untraceable introductions of the disease may occasionally occur, still this cannot be supposed completely to equalize the proportion.

We shall shortly consider the evidence under these three heads:

I. *Do medical and other attendants on the sick show an increased percentage of attack?*

Dr. Copland answers in the affirmative. As far as India is concerned, he adduces 3 instances of positive evidence, viz. the case of the 65th Regiment alluded to by Drs. Burrel and Craw, and the statements of Drs. Stokes and Daun. There are a few other statements of a similar kind in the Indian Reports which he has not noticed. The negative evidence from India, opposed to this view, is very definite and conclusive.

In the 'Bombay Report,' published in 1819, we find 39 letters, of which 37 are from different medical officers. Of these, 23 do not allude in any way to the question of contagion, and mention nothing about their hospital attendants; 6 believe in contagion, but of these, 3 mention that the assistants and hospital attendants were not attacked, viz. Dr. Taylor (p. 195), Dr. Jukes (p. 172), and Captain Sykes (p. 118). Of the other 3 who profess their belief in contagion, 2, viz. Drs. Burrel and Craw, cite the same instance of hospital attendants being attacked, and the remaining gentleman, Mr. Coats, does not refer at all to hospital attendants. (Bombay Report, p. 145.)

In the 'Madras Report,' published in 1824, we find altogether 8 instances where the reporters state that those nearest the sick were attacked, but several of these are very doubtful cases, being imperfectly and loosely reported. The greater number of reporters do not make any statement on one side or the other. The following gentlemen expressly state that none of their attendants were ever attacked, viz., Messrs. Gibson (p. 1), Haines (p. 32), M'Andrew (p. 33), Smith (p. 38), Sutton (p. 41-5), Scott (p. 57), White (p. 65), Campbell (p. 77), Goldie (p. 137), Mitchell (p. 141), Chalmers (p. 142), Fasken (p. 201), and Barton (p. 208). In addition, Mr. Scott, the editor of the Report, brings forward many other cases "of the most striking instances of immunity from the disease under the most intimate personal intercourse." (Madras Report.)

In the 'Bengal Report,' published in 1820, we find the editor, Mr. Jameson, stating, "that the medical observer, in his attendance upon patients, did not find himself or his assistants more liable to be attacked by it than such persons as had no communication with the infected" (Bengal Report, p. 123); and he also gives a list of between 250 and 300 medical men in the Company's service in Bengal, most of whom saw the disease largely, and of these only 3 persons were attacked, and only 1 attack was fatal.

The chief Indian writers on cholera since this date, who allude to this point, are Messrs. Orton, Annesley, Twining, and Corbyn; and there are two or three allusions to it in various numbers of the 'Calcutta Transactions,' and of the 'Madras Medical Journal.'

Of these gentlemen, Mr. Orton was the only one who believed in contagion, and yet, after examining into the evidence on the point in question, he says:

"We are *therefore forced* to the conclusion, however at variance with the common laws of contagion, that in this disease, at least, in India, the *most intimate intercourse* with the sick is not in general productive of more infection than the average quantity throughout the community." (Orton on Cholera, 2d Edition, p. 326.)

The other writers are all disbelievers in contagion, and make strong statements respecting the exemption of hospital attendants. For these, see Annesley (Sketches, &c., p. 244), Twining (On Diseases of Bengal, p. 536, 1st Edition, 1832), Corbyn (On Cholera, p. 96), and the 'Calcutta Transactions' (vol. v, pp. 318-19, and vol. vii, p. 330).

The negative evidence then, as far as India is concerned, is exceedingly strong, and did our limits permit us to extract the different statements on either side, it would be seen how much more definite and satisfactory are the arguments of those who argue for the immunity of the hospital attendants, than of those who hold the opposite opinion. In Egypt, Clot Bey states that no hospital attendants were attacked. (Annales de Médecine Physiologique for 1833.)

The European evidence on this point is more contradictory, but still the negative side has a great preponderance of opinion and statement. At Orenburg the attendants were entirely exempt. (Lichtenstadt.)

In St. Petersburg it appears that there was from some cause an unusual mortality among the physicians, but it is not known whether all those who died of cholera had been in personal contact with the disease. Drs. Russell and Barry think that many hospital attendants were attacked in the ill-ventilated hospitals. In some hospitals very few were attacked. Dr. Hamett, on the authority of Drs. Barchervitz and Daun, denies that any of the nurses or sick attendants were attacked in the Cholera Hospitals of Dantzic, Moscow, St. Petersburg, and West Prussia. (Hamett's Report on Cholera at Dantzic, p. 157.)

Sir G. Lefevre "never knew any instance of sick persons communicating the disease to their attendants." (On Cholera in St. Petersburg, p. 33.)

Dr. Becker states that hospital attendants were attacked in Berlin, but his evidence is not satisfactory. He quotes a number of instances in the Cholera Hospital, No. 1, of Berlin, as having been affected by cholera, but on examining these it is extremely doubtful whether more than 4 out of 39 were cases of true cholera.

In Warsaw the medical men and attendants enjoyed a remarkable exemption from attack. (Relation Historique, &c., par A. Brierre de Boismont, p. 130.)

In Paris the physicians and surgeons of the Hôtel Dieu, which received a great number of cholera cases, signed a declaration that they had not observed any circumstances authorizing them to suspect contagion. This of course implies the immunity of the attendants. To this declaration are appended the names of Dupuytren, Gendrin, Breschet, Majendie, Petit, Recamier, and others of equal celebrity.

Without wearying our readers by undue prolixity, we may state that the negative evidence afforded us by the spread of the cholera in Europe, preponderates greatly over the positive evidence, though not perhaps in so great a degree as the Indian evidence on the same side.

We do not see how we can avoid the conclusion, that there is not sufficient evidence to prove that those nearest the sick are more liable to attack than others not so exposed.

II. *Do individuals or bodies of men, in carrying the disease from place to place, communicate it to the fixed inhabitants?*

Unless a disease be virulently contagious, it is exceedingly difficult to decide a question of this kind. In addition to all the sources of fallacies attributable to variations in the morbid agent, or to the systems on which it acts, we have to take into account the partial and biassed evidence always given on either side of a disputed question, the possibility of coincidences, the doubts which must always exist as to the correctness of diagnosis, and other difficulties of a similar kind.

The carriers of the contagious disease may either be single individuals, or bodies of men, either military or civil. The movements of troops are those best fitted for our examination, as the men move more compactly, and there is no doubt of the time when they enter or leave a place. Bodies of men, not military, are scattered over the country, enter towns and villages at indefinite times, and it is very difficult to prove that the disease was not antecedent to their entrance.

We shall therefore confine ourselves chiefly to military movements, and shall select for criticism two instances of the supposed conveyance of cholera by detachments of troops. The full discussion of all reputed cases would exceed our limits.

1. The first instance is the presumed transmission from Nagpore to Jaulnah in 1818. This is a strong contagionist case, and we have selected it because it is quoted not only by both Drs. Copland and Kennedy, but also by the majority of writers on cholera, and among them the very able author of the article "Cholera," in the 'Cyclopædia of Medicine.'

We possess three Reports from Jaulnah at this time, viz. from Mr. Peyton, the staff-surgeon; from Mr. Campbell, the assistant-surgeon of the Horse Artillery; and from Mr. Kellie, the assistant-surgeon of that native corps.

This last gentleman is a contagionist, and it is from his report that this instance of conveyed cholera has been derived. Dr. Copland, in common with many advocates of contagion, overlooks altogether the reports of Messrs. Peyton and Campbell, who both decide against contagion, and who, with opportunities of observation equal to those of Mr. Kellie, are at least as worthy of being consulted.

In the report from Nagpore it is merely said "to have originated among the citizens in the middle of May, but no case occurred among the troops till the 26th or 27th, although our Sepoys were in habits of daily and intimate intercourse with the inhabitants." (Madras Report, p. 65.)

Jaulnah is but a short distance from Nagpore, and of course, in the gradual progress of cholera, it might be expected to arrive at the former place. The south-west wind however prevailing, its progress was slow, and it took six weeks to traverse these few miles. During this time there must necessarily have been almost daily arrivals of natives from Nagpore, as no quarantine was ever dreamt of. If there were such arrivals, and it is the duty of the contagionist to prove there were not, they failed to propagate the disease. But let us hear Mr. Kellie :

"Several heavy showers fell about the middle of June. The disease prevailed in Nagpore during the month of May, and upon hearing of the march of Captain Doveton with a detachment, some of whom were affected with the cholera morbus, it was generally apprehended that the disease might be brought hither with it. The detachment arrived here towards the end of June. The cholera appeared on the 3d of July." (Madras Report, pp. 70 et seq.)

To make his case good and prove something beyond coincidence, Mr. Kellie should have shown, if possible, the first steps and commencing spread of the contagion. No care, however, seems to have been taken in inquiring into particulars, and consequently we must receive Mr. Kellie's statement with some degree of doubt.

This doubt is, however, still more increased as we proceed; we find Mr. Peyton the staff-surgeon, denying the contagion, and attributing the disease to the peculiarity of the weather, the features of which he details in his Report. Mr. Campbell makes similar observations. So that here is conflicting evidence : are we to believe Mr. Kellie or Messrs. Peyton and Campbell? The only way, supposing the opinions of all to be of equal weight, is to fall back on general experience, and inquire whether similar instances afford support to one or the other party.

We are not certain, however, that Mr. Kellie is entitled to much credit. He had evidently prejudged the case, as appears by his anticipating that the disease would be brought by Captain Doveton's detachment; and he makes one statement which is certainly erroneous. He mentions that during the epidemic, the "Royals suffered much," and the "Horse Artillery little," and explains this by saying, that the men of the first corps had intercourse with infected people, and those of the second not.

This is, however, contradicted both by Messrs. Peyton and Campbell.

The sufferings of the "Royals" are attributed by Mr. Peyton to their being in "old uncomfortable barracks," and he says when they were moved into tents, the number of attacks was diminished one third in a single day. He expressly mentions that no check was put on communication.

Mr. Campbell's evidence is still more important, and his position as medical officer of the troop of Horse Artillery referred to, enabled him to speak with great decision on this point.

"That this disease is not infectious, but is caused by the direct application to the surface of a peculiar miasma, existing in particular strata of the atmosphere, has been fully proved by the history of its progress, as well as by the phenomena attending it. Had it been infectious at this station, the Horse Artillery could not have escaped with so few cases, situated as *they were in constant communication with the Royals, and the bazaars of the force.* Neither, had it been infectious in its nature, could the medical officers, and the numerous attendants on the sick, who must have been peculiarly exposed to infection, have escaped its ravages." (Madras Report, p. 77.)

Here is direct evidence that Mr. Kellie made an unfounded statement, when he attributed the exemption of the Horse Artillery to want of intercourse. The contrary is positively asserted, by the medical officer of that corps.

In fact, this celebrated instance and reputed proof of contagion is clearly untenable; yet this is the strongest example to be found in the whole of the Madras Reports.

We have carefully reviewed the whole history of cholera breaking out in the marches of troops in India, as far as such history is known in the various reports published in the Presidencies, and in the different writings of authors and periodicals, and we can find no unequivocal instance of transmission. Mr. Jameson satisfactorily accounts, in our opinion, for the attack of the disease in the two doubtful cases detailed in the 'Bengal Report.' Mr. Scott gives 50 marches made by European and Native troops in 1819, 1820, and 1821, and yet we can find only 4 instances in which such marches were supposed to have carried the disease. Dr. Lorimer in his excellent Report, in which 144 marches with cholera are recorded, is able to contribute little evidence on this point; he says merely "in 6 instances it is incidentally recorded to have broken out among the villages." (Lorimer, p. 8.)

In any future observations which may be made on marching in India, we would recommend attention being particularly directed to this circumstance; and the commanding officers, and surgeons of regiments, should be called upon to state their reasons for believing that the outbreak of cholera in a village was really posterior in time to the passage of troops, and not contemporaneous or even slightly anterior.

As an instance of apparent coincidence in the attack on troops and towns, through which they march, we would instance the march of H. M. 69th Regiment in 1818, to Cannanore. The regiment was attacked with cholera on the banks of the Madoor River, 7 miles from Seringapatam; the disease prevailed severely as they marched through the Wynaad Pass, and remained till they arrived at the hill-pass of Pereapatam. The villages and towns on the route were affected either at the time, or subsequently to the passage of the regiment; consequently Mr. Gibson, the assistant-surgeon, unhesitatingly refers it to contagion. But a stricter survey of the mode in which cholera spreads, renders it doubtful whether this may not have been a mere coincidence; the disease may have been creeping along in its usual singular manner, and making, as it were, equal marches with the troops. And this really appears to have been the case. After descending the Ghauts, the regiment seems to have outmarched the disease, and in November, 1818, entered Cannanore healthy, after having been put in quarantine. The cholera, however, followed them on the same route, and attacked Cannanore, after the 69th had been there a length of time sufficient to remove from them all suspicion of contagion. The account of this march also gives us an illustration of the way in which evidence may be vitiated by time. Mr. Gibson, the assistant-surgeon of the regiment, detailing this march in the 'Edinburgh Medical and Surgical Journal,' (vol. xxxviii, p. 289,) states that cholera did not reach Cannanore for a year after the entrance of the 69th Regiment, and then came down coastways from Bombay. This is an error of memory, as Surgeon Wyse reports it both at Tellicherry and Cannanore, in December 1818, and January, 1819 (Madras Report, p. 147); and Mr. Scott states that it commenced at Cannanore, on the 5th of December, 1818. (p. 11.)

2. The second instance we shall adduce of troops apparently conveying cholera, is an European case; viz. the presumed introduction of cholera into Poland, by the Russians. We have taken this case because it is cited not only by Drs. Copland, Becker, and others, but has been supposed generally to be a decided instance of transmission.

"The cholera morbus," writes M. Boisseau, "declared itself in Poland the 10th of April, 1831, at Iganie, after a battle with the Russians. This circumstance has been considered manifestly to demonstrate the contagion and the importation of this disease." (Traité du Choléra Morbus, &c., par F. G. Boisseau, Paris, 1831, p. 188.)

But afterwards M. Boisseau says, "already, in 1830, M. Sauvé had observed it with the same symptoms." (Ibid. p. 188.)

M. Brierre de Boismont was present at the combat of Iganie, on the 10th of April, and informs us that the first deaths took place among the Poles on the 13th. He discusses the question of contagion very fully. He says that the troops encamped constantly on a marshy ground, and drank muddy and unhealthy water. The divisions of the army which drank this water were those chiefly attacked. (Relation Historique, p. 137.) He describes vividly the miserable condition of the Polish army, and points out how predisposed they must have been for the attack of any disease, contagious or otherwise.

"Conceive, " he says, " these men, wan, ghastly, yellow, and emaciated, whose features express their sufferings, enfeebled by long marches, by privations of every kind, bivouacked for five months, in bitter frost, in woods or on marshy soils, and one can with difficulty form an exact idea of the state of these unhappy victims of war." (Ibid. p. 5.)

" After the battle" he adds, "the troops returned to their former bivouac, and on arriving, they drank eagerly of the muddy water of the marsh." (Ibid. p. 138.)

He seems inclined to think these circumstances would explain the attack of cholera, but does not hazard a decision, and concludes his examination by saying, that immense difficulties encompass the subject which cannot yet be solved. (Ibid. p. 143.)

The question then seemed very doubtful to an eyewitness, who would have been able to come to some conclusion, if this had been attainable.

The French Commissioners sent to Poland by the Minister of War, especially to examine this occurrence, also leave it undecided, but express themselves more opposed to contagion than in favour of it.

"The contagion of cholera morbus is not yet proved in Poland. (Chamberet.) It is better to remain doubtful on this question." (Trachez.) (Du Choléra Morbus de Pologne. Renseignemens sur cette Maladie &c. &c., par la Comm. envoyée à Varsovie par M. le Maréchal Duc de Dalmatie, Paris, 1832.)

The Commission also records a remarkable fact.

"Nevertheless, two months before this time (the battle of Iganie), and before there was any question of the appearance of the cholera in Poland, Dr. Brandt, the President of the Committee of Health, had observed all the symptoms of this formidable malady in three individuals." (Ibid. p. 14-15.)

The Commission afterwards remarks:

"We have shown that the cholera, which we have observed in this country, does not communicate itself from one person to another either by contact or infection." (Soit par le contact médiat, soit le contact immédiat.) (Ibid. p. 57.)

With evidence so conflicting and various, it is impossible to feel that this supposed introduction into Poland is a case on which we can safely rely.

These two examples must be taken as samples of the uncertainty, which surrounds every similar case.

The instances in which individuals have been supposed to introduce cholera into towns or districts are very numerous. Yet in no one case is the evidence perfectly unequivocal. This is so much the case in India, that Mr. Orton, although a believer in contagion, is compelled to write—

"Another hiatus in the evidence of contagion is, that though the importation of the disease has so often been found immediately to precede its appearance in the inhabitants of a place, and even the first cases to arise in the neighbourhood of the imported virus, it has scarcely ever been possible to trace these cases in anything like a regular series." (Orton, p. 326.)

Mr. Scott's case is the strongest in India. Strong doubts can be thrown on that quoted by Dr. Taylor, in the 'Bombay Medical Report.'

Let us take a few individual cases out of India. Its introduction into Orenburg, in 1829, is ascribed either to the caravans, or to the Kirghis Kaisacks. But the Report of Dr. Lichtenstadt seems to us conclusive against either supposition. (Edinburgh Medical and Surgical Journal, for 1831. No. 108, p. 121.)

In the case of Astrachan a most important item of evidence is omitted; as every one will see on reading over the evidence. The ship supposed to have imported the disease was still in the lazaretto, when 17 days after her arrival the disease broke out in the town. No attempt even is made to prove intercourse between the sailors and the inhabitants.

So also at St. Petersburg, Drs. Russell and Barry state that "no direct intercourse has yet been traced between any of the first 5 or 6 cases." (Report, p. 25.) We cannot indeed conceive how introduction from vessels arriving from infected places on the Volga, can be maintained, when we find the first case in St. Petersburg occurring in a journeyman painter, according to the Russian medical men (Protocol sur le premier malade, p. 30), who had not been out of the city, nor had intercourse with any diseased person. Drs. Russell and Barry indeed state that a merchant, who was in his ship in the river, was first seized, but they admit this was a slight case, and out of danger in two hours; and independent of the doubt which must be felt as to whether so mild a form could transmit the disease, no communication was traced between the merchant and the painter.

Jannichen states that at Moscow the disease could not be traced to importation. Mr. Fergus makes a similar avowal as to Vienna.

Another case which has been much quoted is that of the Topaze frigate, which arrived at the Mauritius from Ceylon on the 29th of October, 1818, having lost 3 men from cholera; 20 days after her arrival cholera appeared among the natives in the bazaar. Without going into this disputed question, it appears to us that the long period of incubation we should have to assume, if we allow the importation by the Topaze, is sufficient to render this case very doubtful. Eight days is the longest incubative period any writer has ever allowed. The Genoese medical commission sent to Hungary make the longest period 5 days. James Kennedy makes it 3 days. (On Contagious Cholera, p. 215.) Dr. Becker, of Berlin, makes the extreme period 6 days. (On Cholera in Prussia, p. 33.) Russell and Barry make the extreme 5 days. (Report, p. 93.) The 'Cholera Gazette' gives 7 days as the longest period.

Unless it can be clearly proved that the incubative period is sometimes 20 days, or even in this case longer, as it would have to be calculated from the date of the last death on board the frigate, we cannot admit this case as an undoubted and unequivocal instance. The able Report of Dr. Kinnis supplies us with other strong reasons against the importation, but considering the above argument as conclusive, we shall not occupy space in their detail.

The introduction and the diffusion of cholera in England has been a matter of great dispute. After an attentive investigation of the Sunderland controversy, we cannot consider the introduction into that place as at all proved, or even rendered probable.

In the subsequent spread of the disease there is more proof of transmission. Dr. Simpson, in the 49th vol. of the 'Edinburgh Medical and Surgical Journal,' has collected many very strong instances of apparent contagion; only a minority of these, however, are from his own observation. The death of the two nurses at Barthgate is to us the strongest instance he brings forward.

Mr. Sharkey, in the 16th volume of the 'Dublin Journal,' adduces other similar instances, and Dr. Graves, in his excellent article in the same Number, which contains an able exposition of the chief arguments for contagion, has given evidence of a similar kind.

On the other hand, in certain suspicious cases, a strict investigation has shown the evidence for contagion to be worthless. Dr. Craigie's observations on the cases in Musselburgh show how cautious we ought to be in accepting these instances of contagion, which, till carefully examined, appear so strong and incontrovertible.

In America the disease appeared 8 months after its first outbreak in Sunderland. Dr. Jackson's observations on cholera in Quebec seem to discountenance the idea of importation, and certainly there is no evidence of any emigrant ship, with cholera on board, having arrived at Quebec at the time stated. Dr. Graves, however, is not satisfied with Dr. Jackson's objections.

A much stronger instance than this is to be found in the case related in the 'American Journal of Medical Science' for 1833 by Dr. Dickson. The brig Amelia, with an infected crew, being wrecked on Folly Island, near Charlestown, cholera was communicated to certain persons who came in contact with the diseased crew, but to these persons only, and did not spread in Charlestown. This is, perhaps, the best authenticated instance of importation in the whole history of cholera. Dr. Keckeley, in the 14th volume of the same Journal, attempts to explain it away, but it must be confessed he does not at all succeed in the attempt.

We could ourselves state two or three instances (out of hundreds of an opposite kind) which presented all the ordinary evidences of propagation by contagion.

It may be concluded, then, that the evidence for the contagion of cholera derived from this source is of a much stronger character than that derived from any other quarter.

III. *Do methods of isolation and quarantine ensure exemption in individuals practising them?*

The evidence on this point is completely on the negative side.

Quarantines failed in the only instance in India in which they were tried, viz. at Travancore; they failed at Alexandria and Constantinople. (Clot Bey, and Russell and Barry, p. 67.) They failed in Hungary, Austria, Prussia, and Russia so completely, that Dr, Becker, a strong contagionist, is compelled to state that the inefficacy of the preventive measures in arresting the ultimate progress of the disease is a fact which he willingly admits. (Becker, p. 29.)

Astrachan was in strict quarantine when the disease broke out. A rigid cordon was formed to prevent communication with Russia and Poland, and yet the disease appeared immediately at Dantzic. (Hamett, p. 14; Becker, p. 43.) Konigsberg was attacked in the same way, and in both these cases, as in that of Hungary and Dublin, cholera broke out without any previous communication with an infected place. Dr. Copland states that no instance of such an occurrence is known, but this appears to us erroneous.

The course of the cholera through Egypt affords us striking instances of the inefficacy of quarantines. The harems in Mahomedan countries may at at all times be said to be in a state of quarantine; the seclusion is so rigid and so guarded, that even in the prevalence of the plague, little more precaution can be adopted. In Broussais's 'Annales de la Médecine Physiologique' for September, 1833, is an account from Clot Bey, who states that the harems watched with the most strict quarantine were not exempt. This observer also states that the Viceroy embarked on board a vessel at the first outbreak, and preserved a very strict quarantine; in spite of this, several of his retinue died, and he changed his vessel several times. Clot Bey also mentions that 500 Bedouins encamped in the Desert, and preserving a strict quarantine, did not escape.

In Egypt, as in India, the remarkable fact was noticed that some villages remained altogether free, although there was a constant communication between them and infected places.

On the other hand, we have some instances of cordons and quarantines arresting the disease. The case of the Isle of Bourbon is the most celebrated of these. Cholera was prevailing in the Mauritius in 1818, and Bourbon was accordingly put in strict quarantine. The disease, however, appeared at St. Denis, and was said to have been introduced by the crew of a smuggler. St. Denis being put in quarantine, the disease did not spread over the island. Madagascar, however, being attacked and no quarantine being observed, the mortality was very great.

This is certainly a strong case, but there are some objections to its reception as good evidence. It is derived entirely from the reports of the French physicians, who were bigoted contagionists; the principal writer who insists upon it is M. Moreau de Jonnès, whose elaborate account of cholera is, in many respects, very inaccurate; in fact, so inaccurate, that we cannot be too cautious in our investigation of his statements. He seems to think that nothing is necessary to prove importation of cholera beyond the mere fact of communication existing between two places, one of which is infected. The minutiæ of evidence are altogether disregarded by him; and occasionally, to prove his point, he makes unfounded assertions. Thus he states that Bankok received cholera by trading-vessels from India. Dr. Copland repeats this after him. The fact is, on the contrary, that cholera passed overland, both into Siam and China, first traversing Tartary and Burmah. (Calcutta Transactions, vol. i, p. 205; Bengal Report, &c.) Nevertheless we should have been willing to receive the statements of the French physicians respecting Bourbon, if all subsequent experience had not so completely proved the inutility of preventive measures. If quarantines answered so well at Bourbon, how was it they failed in Europe? The Hungarian cordon was most strictly kept; human intercourse was impossible, and yet cholera appeared. So also in other instances. We agree with Mr. Orton on this point, who says, "we know how warmly the French physicians espoused the cause of contagion, and, therefore, their general statements must be received *cum grano salis.*" (Orton, p. 334.)

The other reputed instances of exemption by aid of quarantine, are unworthy of notice.

The instances of villages escaping is no argument that this was owing to seclusion, as we know that cholera, in its strange and eccentric career, constantly passes along particular tracts of country, omitting, in a manner apparently capricious, some portions of it, and attacking others. An instance of this is recorded in the 'Bombay Report,' and is quoted by Dr. Kennedy. Some villages on the island of Salsette during the first visitation of cholera remained free; this was attributed to isolation: but a few weeks afterwards the disease suddenly retraced its steps, and now attacked the localities it had formerly spared. (Preface and Appendix to the Bombay Report.)

Another remarkable instance is recorded in the 'Bengal Report.' Sundeep, an island in the Sunderbunds, remained, in 1818 and 1819, perfectly free from cholera, although the islands all round it, similar in position, cultivation, and geological features, suffered frightfully, and for long periods, from the disease. This singular and unexplained exemption would doubtless have been attributed to quarantine, if this had been practised, but free and uninterrupted intercourse was carried on the whole time between Sundeep and the adjoining islands.

Another instance is the singular exemption of the island Kristofsky, which communicates with the island of St. Petersburg by two magnificent bridges, and with the town by a thousand barges continually plying; it is a frequent resort of the citizens, particularly on Sunday; yet during the prevalence of cholera in St. Petersburg not a single case occurred here. (Observations sur le Choléra Morbus, par l'Ambassade de France en Russie. Paris, October, 1831.)

Before concluding these brief remarks on the evidence of the contagion of cholera, we must allude to another opinion expressed by Dr. Copland, and which we think he is perfectly consistent in holding. He asserts that "the introduction of cholera into this country was certainly owing to the clothes and bedding of sailors who had died at Riga. Of this fact several proofs of a most incontrovertible nature were furnished me by two masters of vessels." (Copland, op. cit.)

In the absence of knowledge of these "proofs," with which Dr. Copland has not favoured us, we have been able to find no evidence of this fact, and we observe that it is doubted by many staunch contagionists.

Thus Dr. Becker, of Berlin, writes:

"There is in the whole history of cholera no authenticated case in which the contagion has been transmitted from one place to another by letters, new clothes, or merchandise of any description." (Becker, p. 35.)

Doepp, the physician to the Foundling Hospital, St. Petersburg, and a strong contagionist, writes:

"I have given myself great trouble to ascertain if the clothes and linen covered with the perspiration of the sick were capable of transmitting the contagion, but I could not meet with any instance of it." (Doepp, quoted by Russell and Barry, p. 146.)

The committee of St. Petersburg having first decided that clothes could carry the disease, reversed this opinion on further inquiry, and, by an imperial decree of August 1831, the precautions of fumigating goods and other safeguards previously adopted were abolished.

In England, the same opinion was held by the best observers. Dr. Brown states that medical attendants never conveyed the disease in their clothes to their families or patients. (Cyclopædia of Practical Medicine.)

The preceding brief sketch is a faithful sample of a much more extensive investigation which we have made, but which our limits will not permit us to detail at length. We have endeavoured to judge each portion of evidence with the utmost impartiality, and to attach to each observation that degree of credence which its greater or less preciseness and exactitude seemed to demand.

Having arrived at the end of this part of the inquiry, we will state the following as the conclusions, which appears to us warranted:—

1. The evidence for the contagion, in the restricted meaning of the term, of cholera is, for the most part, imperfect. This remark particularly applies to the observations collected under the first and third heads:—exposure to the disease is not proved to be followed by increased liability; and isolation is not proved to bestow increased security.

2. At the same time, the instances where cholera has appeared in a place after the arrival of infected persons, are very numerous, and seem to indicate something more than a mere coincidence. There are also some authenticated instances, in which people who had been in contact with these newly arrived persons were the first, or the only sufferers.

3. Admitting that there is evidence of contagion in a limited degree, and in certain cases, it is evidently impossible to consider this a sufficient explanation of the way in which cholera sometimes suddenly attacked large bodies of men, or has travelled over the world, and still continues to affect immense tracts of country in Asia.

In fact, we do not think any one can study the peculiar mode in which cholera attacks a country, either as detailed in the voluminous writings on the subject, commencing with Mr. Jameson's unequalled Report, or by personal witness of its past invasion of Europe, or its present ravages in India, without feeling that contagion *per se* is utterly inadequate to account for its attendant phenomena.

The question then is, whether these two conditions are compatible: if we can conceive a disease to be ordinarily propagated by other agency than that of reproduction by the human system, and yet at times to be so reproduced, and to acquire, in ordinary language, a contagious property?

This is a compromise between two extreme opinions, and is not, perhaps, likely to find favour with either party. Its very simplicity is against it; it seems like cutting the Gordian knot; it may, nevertheless, be the truth.

The observations made at the commencement of this article, on the remarkable changes which the morbid poisons in various ways undergo, at any rate show the possibility of this occurrence. In the present state of science, it would be impossible to prove its absolute certainty.

After all, the question is most important in a scientific view,—practically, as far as legislation and prevention are concerned, it may be considered settled. There can exist no doubt, that even if cholera be contagious, it cannot be localized and restrained by quarantines; theory and experience both demonstrate the inutility of preventive measures of this kind.

The plague, the yellow fever, and the cholera stand nearly in the same position; the discussion of their contagion is in no case yet ended. And we doubt whether a decision in which all parties can agree will be arrived at in these three instances, without the adoption of methods of research more accurate, and of modes of inquiry more comprehensive. The final determination of this question may perchance be the triumph of an advanced organic chemistry, which may hereafter render determinate and visible the agents which are to us so recondite and obscure.

Leaving then, in this indefinite state, the question of the contagious or non-contagious nature of cholera in all instances, let us turn for a few brief moments to the hypotheses which have been formed as to the nature of the agent causing it.

1. The disease has been considered by some to be entirely atmospheric; that is, produced by changes in the ponderable or imponderable elements of the air without the addition of some new ingredient. This hypothesis is contradicted, however, by all the phenomena, which seem decidedly to point out the presence of a virus derived from sources foreign to the atmosphere, and merely existing in it.

2. The materies morbi has been supposed to be a modification of vegetable miasma, produced by peculiar causes of heat, moisture, &c., acting on the productions of the soil.

3. It is supposed to be an agent evolved from the crust of the earth, and produced by volcanic and other changes. This has been a favorite hypothesis in all epidemics, and many curious coincidences between earthquakes, volcanic eruptions, &c., and epidemic diseases have been collected in the extraordinary work of Noah Webster, and in Hecker's 'Epidemics of the Middle Ages.'

4. Another party, leaving in obscurity its origin, regard it when witnessed by us as always allied to the human system. This is the contagious doctrine as developed by Copland, Becker, and others.

5. It has been surmised to be caused by animal life, existent in the atmosphere under certain circumstances.

Our space will only allow us to say a few words on this last very interesting and ingenious conjecture. The arguments on which it is founded have been well stated by Dr. Holland in a chapter which we shall not attempt to imitate, and could not without injury condense. We believe he has stated the principal circumstances which give plausibility to this view. It is certainly an argument in favour of this hypothesis, that it is listened to with greater favour at the present day than it was in 1755, when Linnæus published the essays of Nyander. The progress of science since that time has shown the universal and, apparently, the necessary prevalence of life in all departments of nature. Accustomed as we are to consider everything around us as endowed with life; to recognize in rocks and soils the débris of vitality; in the most subtile fluids the dwelling-places of animalculæ; even in the atmosphere itself the matrix of microscopic germs—there is nothing incredible in the hypothesis of the existence of animals as yet unrecognized by our sight or instruments. Every one even is willing to admit that such races of animals must necessarily exist, and will some day become known to us. But, admitting this, and admitting that the hypothesis of insect propagation being a cause of disease would apparently accord with many of the phenomena familiarly witnessed during epidemics, we cannot allow that any more than an ingenious speculation is made out. The assumptions which we must make before entering on the proper evidence of such a view are enough to stagger our belief.

1. We have to assume the existence of these unrecognized animalculæ.

2. We have to assume that these animalculæ really possess the singular property of producing a formal and peculiar disease in the animal system.

3. We have to assume that for different diseases there is a distinct race of animalculæ. This assumption is quite necessary: it is impossible to restrict the influence of this cause to cholera alone. It must be extended to all diseases, or, if not to all, at any rate to all contagious and epidemic diseases. This, in fact, was done by Nyander, and he has been followed by many writers, particularly by Adam Neale, in his work on 'Animate Contagions.'

Moreover, if this theory be accordant with many of the phenomena of cholera, so also are other suppositions which attribute more to chemical agency than to vitality in the production of the disease. The peculiar powers which modern chemistry is bringing to light are, to say the least, as probable agents as vital influences can be proved to be. It is now well understood that the quantity of a chemical agent is in no degree commensurate with its peculiar effects. A single globule of yeast excites fermentation in a liquid of a thousand times its bulk. A single grain of chloride of palladium injected into the veins produces, according to the interesting experiments of Dr. Blake, an extraordinary effect on the whole mass of blood. It is, perhaps, more in accordance with our present mode of thinking, to attribute epidemics to some chemical virus, which is in too small a quantity to be detected by our present means of research.

But we must leave this ingenious speculation of "insect propagation," and can only wish that we could justly give it some name, more honorable than that of "speculative." It is one of those points which lie on the outskirts of science, which is to be discussed and to be remembered, but not at present to be adopted. The science of medicine may be compared to a vast continent, on which have been founded numerous little colonies, complete in themselves, but surrounded by unknown and unproductive regions. Far off in the interior of the wilderness lie these obscure, though vast speculations; dimly we see them, but cannot discern whether they are real, or but visionary structures, similar to those which the bewildered traveller may sometimes witness, when before his eyes rise the lofty fanes and gorgeous temples of the desert mirage.

We shall conclude this article with a brief summary of the principal phenomena manifested by the cholera or its virus apart from the question of contagion. An immense deal of information has been accumulated on this head, of which we shall only extract the points most certain and most important.

We may review this subject under three heads—1, the virus itself; 2, the state of the media favoring its transmission; 3, the state of the system favoring its reception. We still follow Dr. Holland, in his 'Notes and Reflections' (p. 277), in believing that the whole subject may be comprised under these three heads.

I. *The virus itself.* The first and most striking fact respecting the choleraic virus lies immediately upon the surface. This is, its gradual extension over the face of almost the whole globe. From the moist and level surface of Bengal it passed towards the high table land of Central Asia, the steppes of Tartary, the luxuriant and antique forests of Burmah, and the hot and burning countries of the peninsula of Hindostan. Along the defiles of the Hindoo khosh, and the lower plateau of the Himalaya, across the plains of Persia, and the sandy and untracked deserts of Arabia, it pursued its singular and erratic progress. Penetrating into Russia, it passed gradually over the face of Christendom, unchecked by the cordons and barricades of European civilization. The extent of its ravages is marked by the fact that in almost every language it is named, and, except in Australia and the remote islands of the South Seas, it is known and dreaded by every branch of the great human family.

The consideration of this diffusion is a strong argument in favour of its propagation by human intercourse: but it is only an argument, not a proof. Observers who saw it in India in its outset, such as Mr. Orton and Dr. Russell, and found no evidence of contagion, reversed this opinion after the epidemic of 1830-2. Many indeed, consider this argument incontrovertible. They ask what property it was that could enable cholera thus to traverse nations so diverse and countries so various? What condition in common had the Hindoo and the Englishman, the Chinaman and the American, the Mongolian and the Caucasian? What condition in common had the climates of tropical Asia and of arctic Siberia, the burning regions of the dark Hindoo and the cold and northern latitudes of the Saxon and the Celt? The explanation which most easily and naturally occurs is, that the disease is independent of all climates and of all vicissitudes; that it is not bound to the soil, nor circumscribed by the limits of the winds, but that, from the living frames which testify its presence, it derives at once its origin and its progress.

Next to the universality of its prevalence, the most striking fact about the agent is its peculiar and irregular mode of spreading. It differs in this from every other known disease. Influenza approaches nearest to it, but there are important differences in their mode of diffusion. Both alike seem to be uninfluenced by temperature, and to prevail equally in all climates. But influenza travels much more rapidly, is more like an atmospheric disease, is not so bound to the soil, and in the intervals of its occurrence seems completely to disappear and not to manifest itself in certain places, as cholera has continued to do since 1817; or, if influenza does not altogether disappear, its form becomes modified. Cholera, on the contrary, creeps along, selects a certain tract of country, and shows an indisposition to traverse seas, and even rivers, although it selects the banks of these, while influenza has even been known suddenly to attack a ship's crew in the middle of the Atlantic.

Dr. Kennedy calls attention to the similarity of many of the phenomena of yellow fever to cholera. He says: "Its characteristics (yellow fever) as an epidemic seem in some respects so parallel with cholera, that almost the very same terms may describe both, excepting, indeed, in the grand distinction of contagiousness." (Hartley Kennedy, p. 79.) The alliance of cholera with remittent fever, in point even of symptoms, has been noticed by many writers, and these analogies and resemblances, vague as they are, ought certainly not to be disregarded, as they may point out hereafter the mode of investigation necessary to be pursued.

A great peculiarity about the choleraic virus is, its gradual development and exhaustion. Thus it enters a place and, something in the manner of plague, attacks a few of the most predisposed individuals; then increasing, it rapidly reaches its acme, and then declines. Mr. James Kennedy attributed to it a kind of monthly course; and Mr. Orton and others have fancied the variations were dependent on lunar changes. Mr. Scott's examination, however, does not countenance this.

Dr. Lorimer adds another example to this curious peculiarity:

"The average duration of a severe epidemic outbreak of cholera is under four weeks, and the period of its greatest virulence is limited to about one third part of the whole time of its continuance, or about nine days, during which time nearly one half of the attacks are fatal, while, during the whole period of the epidemic, the deaths amount to one third of the seizures.........The virulence of the epidemic is confined between the fourth or fifth and fifteenth day of the epidemic." (Lorimer, p. 10.)

It must be remembered, however, that Dr. Lorimer is speaking only of marches. In cantonments the attacks are generally shorter than this.

It cannot be said, however, that this course is uniform. Sometimes it attacks quite suddenly, and in a way almost inexplicable. We have known, in a cantonment perfectly free from the disease, ten men of the same regiment to be attacked in a single night, and every one of these cases to be fatal, while neither in this or the other regiments at the same station did any subsequent case occur at this time. In the Marquis of Hastings' camp on the banks of the Sinde, in 1817, it reached its acme on the second day.

In this development and decline, cholera seems to have some singular analogies both to plague and epidemic smallpox.

It is this fact also which has given some countenance to the opinion before referred to, that the development of animal life comes nearest to the spread of the choleraic agent.

The cessation of cholera has been attributed by some to the exhaustion of those predisposed to the attack. One fact seems to countenance this, viz. that troops have entered a camp which cholera has just left, and have then begun to suffer severely from it, while those who were previously in camp have experienced no return. From this absence of predisposition is also perhaps to be explained the exemption one corps will exhibit while marching with another corps which is infected.

That the choleraic agent is highly diffusible and susceptible of alteration is proved by a multitude of observations. Sudden atmospheric changes often destroy it: as often announce its appearance. The Indian records abound in instances of this kind, and to these and to Mr. Orton's work we refer for examples.

II. *Condition of the media favoring the development of the agent.* Although our knowledge of the choleraic virus is so limited, it is not so with the conditions which favour its diffusion. These things are cognizable by our senses, and, accordingly, an immense mass of facts has been collected, which gives us information of the highest importance.

These are contained chiefly in the excellent and truly scientific Report from Bengal, edited by Mr. Jameson, in the 'Madras Report,' which presents us with a great accumulation of facts, and in the work of Mr. Orton. Dr. Lorimer's Report also adds to our knowledge on this subject.

We may consider this subject with reference to—1, the soil; and 2, the atmosphere.

1. It is an universal remark that low, confined, and particularly marshy places suffer more frequently and more severely. This has been the case both in India and in Europe. High and airy situations are visited more seldom and with less severity.

The account which Mr. Jameson gives of the encamping ground of the Bengal army in 1817, affords us, in a few words, the characteristics of the soils which have been found most favorable for the propagation of the virus.

"In the ground of encampment in which the disease prevailed most, the soil was low and moist; the water was foul, stagnant, and of brackish quality, and everywhere not more than two or three feet from the surface of the earth, and the vicinity abounded in animal and vegetable putrified matter; whereas, at Erich, where the army regained its health, the situation was high and salubrious, and the water clear and pure from a running stream." (Bengal Report, p. 106.)

The banks of rivers seem also efficient in promoting the spread of the disease. Its peculiar attachment to rivers, and the way in which it spreads along them, independent, it may be, of human intercourse (Orton and others), is indeed a very peculiar feature, and one almost distinctive of cholera. It is not the mere moisture which aids the development, as the poison often remains close to the bank, and does not attack the crews of ships lying at some distance. Or it passes along one bank without touching the other, although free intercourse goes on, and then, suddenly crossing the river, traverses in an opposite direction the bank hitherto untouched.

In entering a town cholera almost always attacks the lowest and dampest quarter. This was universally the case in India. It was the case also in Persia (M'Cormack, in Medico-Chirurgical Transactions, vol. xii); in Russia, Hungary, Prussia, &c. (Lichstenstadt, Becker, De Boismont, Boisseau, Hamett, Russell and Barry, &c.); in England (Craigie, Brown, Hazlewood and Mordey, and numerous others); and, in fact, this observation is universal.

It does not appear, however, that jungly or marshy country by itself is always the most favorable for development. Dr. Lorimer makes the following remarks on this subject:

"With regard to the nature of the country passed over; this, of course, has been very various, hilly, jungly, and open; but the encamping ground, with few exceptions, has been described as good and open. In connexion with this point the following returns, taken from the tabular statement on the accompanying map, relative to the nature of the country and soil at the place of attack, are given. They show that the greatest number of attacks have occurred on cotton soil, and what is described as open country. Further, it was thought proper to direct attention to the distance of the nearest river from the spot of outbreak, and the results are here briefly but clearly exhibited; 49, or nearly one third of the whole number, have occurred on the banks of rivers, and 82, or fully one half, within five miles." (Report, p. 6.)

Of 121 epidemic attacks, 60 occurred on the "black, or cotton soil," 46 on the "red soil," and 15 on "various soils." (Ibid. p. 6.) In the same 121 cases, 99 occurred in country described as "open," 15 in "jungly country," and 7 in "hilly districts." (Ibid. p. 7.)

It does not follow, however, that cholera will not ascend hills, although these decidedly enjoy a much greater immunity than the plains. For the first three years in India it was repelled by the Himalayas. It afterwards attacked the towns on the lower plateau, and it was a singular circumstance, which after observation has confirmed, that the per centage of attacks to the number of inhabitants was much less than in the lower regions, while the fatality was exceedingly great. (Calcutta Transactions.) It seemed, indeed, as if only those particularly predisposed were attacked, and the majority of these died. We are not aware that cholera has been observed at an altitude of more than 6000 feet above the level of the sea.

Dry sandy soils are unfavorable to its development. This was witnessed both in India and in Arabia. They are not, however, exempt.

That an effect is produced on the virus by emanations from the soil, independent in whole or in part of atmospheric states, is evidenced by the sudden change which sometimes occurs in the choleraic virus by changing the ground of an infected army or battalion.

The army of the Marquis of Hastings had not retreated fifty miles from the banks of the Sinde river before the disease left them. A portion of the army in Burmah being attacked with cholera in 1825, moved to higher ground, only a mile and a half distant, after which not another case occurred. (Orton.) Here they could not have changed the atmospheric condition. Either ascending or descending hills has often checked cholera in regiments. Many examples of this are given both by Jameson and Scott.

Dr. Lorimer remarks:

"In 63 instances, or fully one half of the epidemic outbreaks, it is recorded that the disease continued to the end of the march, ceasing gradually: in 22 instances it is stated to have disappeared on change of weather, or after heavy rain; in 11, after crossing a river; while in 9 it is distinctly recorded that the disease not only continued, but became aggravated, both in frequency and severity, after crossing a river; in 5 instances the disease disappeared on the regiment being encamped on high ground. In the remaining 10 the required information on this point is not recorded." (Report, p. 11.)*

It must be confessed, however, that our knowledge of the effect of soil is limited and of the most general kind. It remains to be seen whether geological features have any influence upon it. That these affect remittent fevers is now known from the observations of Mr. Heyne on the hill forts of India that are subject to or exempt from fevers. It remains to be determined whether cholera is at all influenced by the same circumstances.

2. The conditions of the atmosphere in cholera attacks have been the subject of much discussion, and some have even supposed that the disease is altogether irrespective of them. This is, however, decidedly erroneous, and contradicted by all observations on the subject.

To enter into this extensive subject would exceed our limits; we shall merely indicate the most prominent facts. We refer our readers to the 'Madras Reports' and to Mr. Orton's excellent work, and more particularly to the 'Bengal Report,' pages 149 to 165, and pages 106 to 122, where is recorded an immense mass of evidence on the effects of low, damp situations, filth, drains, stagnant water, rivers, and changes of temperature, moisture, barometrical changes, on the diffusion of the disease.

It may be true that the choleraic virus will prevail independent of these, but that it is influenced by them it is impossible to doubt.

Temperature by itself seems to have little effect on the virus: neither the heat of Indian or the cold of the Russian winter retard its propagation. It also prevails in India in the cold and the hot months. Sudden changes of temperature often attend its outbreak, but then other causes are generally concerned in the production of these, which may have an effect on the virus.

The humidity of the atmosphere has attracted a great deal of attention. The outbreak of the disease in the provinces of Chittagong, Behar, Dhacca, and Sylhet was preceded and attended by unusual floods in Bengal (Jameson's Report, Introduction, pp. 68 et seq.; Tytler, on the Morbus Oryzeus.) The seasons in 1816 and 1817 were indeed so remarkable, as to have attracted the notice of every one, and to "have formed the topic of common conversation." (Jameson.) During the progress through

* The virus seems, indeed, almost to adhere to the soil. It seems to prefer travelling overland. Thus, in 1818 it passed down to Palamcottah, at the very end of the peninsula, before it reached Ceylon, although between Calcutta, Madras, &c., and Trincomalee there was constant communication. So also it took five months (three months—Orton) to pass from Masulipatam to Madras, and crept during this time very slowly along the land, while there is a constant passage of native boats between the two places, and the passage only occupies ten days.

India the outbreak of the disease in almost every place was attended by phenomena similar to those which attended its primary appearance. (Orton.) Orton traces this progress with great care. (pp. 168 et seq., 2d edition.) In Europe the same thing was noticed by several observers. Jannichen of Dresden decided that the choleraic virus had a peculiar affinity for moisture. Baumgartner, on the other hand, could trace no hygrometrical alterations during its prevalence. It broke out in Russia in one or two places when the thermometer was—20° Fah., and when the air must have been nearly dry.

In India it sometimes appears at the commencement of the monsoon; occasionally, on the other hand, a very heavy fall of rain checks it. Messrs. Jameson and Scott remark that troops marching in dry and cool months enjoy a considerable immunity from the disease.

Dr. Lorimer remarks, that "in the cool and dry, and hot and dry weather the ratio of attacks on the march is nearly the same, and in the cold and rainy, and hot and rainy weather the ratio also corresponds to an unit, though it will be seen, on reference to the tabular statement, that in rainy (whether cold or hot) weather the ratio of attacks is nearly doubled." (Report, p. 5.)

From the table (p. 6) it appears the per centage of attacks to marches per cent. is, in cool and dry weather, 17·54; in hot and dry weather, 19·11; in cool and rainy weather, 35·95; in hot and rainy weather, 35·84; and in variable weather (that is, "cold, hot, and rainy"), no less than 68·42.

In the 'Madras Reports' (published in 1824) the kind of weather is often described as cloudy, close, sultry, with drops of rain, or occasional showers. Mr. Orton says, there is no instance known to him of it breaking out epidemically during the early years of the epidemic "in the serene or settled weather." (Orton, p. 184.) It would appear, however, that it has often been present in weather which is described as "exceedingly pleasant," although this might be from occasional showers lowering the temperature.

That moisture *per se* is not powerful in spreading the disease may be presumed from the retardation of the virus by seas and broad rivers, but it does not follow from this that moisture may not be one of the conditions which is necessary to constitute the peculiar disposition of the air necessary for the rapid development. It is certainly in this direction that we look for some probable elucidation of the unknown laws of the choleraic virus.

The winds retard and aid the passage of the agent. It is a common, though an erroneous notion, that cholera generally passed against the wind. This is true only to a certain extent: a strong adverse wind always delayed its progress, although it did not prevent it.

This may be instanced by the case we have already cited of its first passage from Masulipatam and Madras. From Masulipatam it passed very slowly in the teeth of the wind; but from Madras, the north-east monsoon setting in, it acquired a great velocity, and rapidly reached the towns and districts to the south, although, on account of the heavy rain, intercourse was interrupted and at times quite arrested.

The idea that it travelled only westward is now known to be a mistake. At the time it was spreading from Bengal towards Bombay, it was also passing easterly through Tartary and Burmah. Of late years it has constantly passed from Hindostan, through and across Burmah, to Siam and China.

Mr. Jameson, remarking that the disease at its first origin had a singular tendency to travel westward, says: "Upon reference to the various reports of the rise of the disorder in different parts of the country, it was discovered that, in a vast majority of instances, the wind was blowing from the east and south-east at the time of its breaking out." (Bengal Report, p. 98.)

Under this head, viz. alterations in the media favoring the transmission of the agent, we are disposed to put the well-known observations of the predilection of cholera for all places where human beings are thickly crowded together. This was particularly insisted upon by Jameson, in a passage extracted by Dr. Copland. The emanations and effluvia from a crowded camp or city seem to give the virus its conditions of existence in a high degree. It is generally supposed that this is confined to large assemblages of men. Dr. Lorimer, however, is inclined to think that our view of this point should be enlarged. During marches, even when the force does not exceed a company or two, he finds "the ratio of attacks decidedly greater as the strength of the regiment increases." (p. 4.) It seems to increase, even with these small numbers, in a very regular manner. Thus it appears that the ratio of attacks to strength per thousand are as follows: for 100 to 300 men = 61·616; 300 to 500 men = 68·085; 500 to 700 men = 58·117; 700 to 900 men = 89·009; 900 to 1100 men = 86·212; 1100 to 1534 men = 132·151. (p. 38.) Treasure parties, which are small, are seldom attacked: out of 352 marches cholera appeared 8 times. (p. 4.)

This is a most important observation, inasmuch as, in ordinary marches in quiet districts, a remedy can easily be found by dividing the troops into small bodies, as is recommended by Dr. Lorimer in his Report.

The electrical conditions of the atmosphere have been supposed to exert an influence over cholera. We might certaiuly anticipate some influence, from the universal diffusion of electricity and its high importance in the economy of Nature, but at present this is mere conjecture. Cholera is said to prevail both when the atmosphere is negatively and positively electrical, so that we have not even the slightest observation in favour of the presumed influence of this agency.

Dr. Prout noticed an increase in the weight of the atmosphere during the prevalence of cholera. This observation at present stands alone.

III. *Condition of the individual favoring the reception of the agent.* Certain pre-existing diseases seem to give a great predisposition for the reception of the choleraic virus. A peculiar kind of watery diarrhea is sometimes seen to prevail in a place where it is raging, and in many cases on this cholerine, as it has been termed, true cholera supervenes. Many suppose this to be the first stage of the disease. Common tropical dysentery does not appear to predispose to it, in any great degree, as there are often many dysenteric cases in hospital, when a regiment is attacked with cholera, and there is no unusual prevalence among them.

Some diseases of the lungs have been supposed occasionally to have a predisposing influence, particularly chronic bronchitis, and pulmonary emphysema, but the observations on this point are not conclusive.

The action of mercury affords no immunity; many examples are recorded in the Bombay and Madras Reports, and in later publications, of patients freely salivated being attacked.

Debility and impaired health from any cause are predisposing circumstances; but it cannot be denied that many men are attacked in the plenitude of health and strength.

The influence of marching, in India, was much insisted on by Orton, who devotes a chapter to the special consideration of this subject. His conclusions are completely borne out by Dr. Lorimer, whose tables are very conclusive. The ratio of attacks to marches per cent., in all branches of the Madras native service, is as follows. Under 200 miles the ratio of attacks is 8·52 per cent.; under 400 miles it is 33·94 per cent.; under 600 miles it is 33·33 per cent.; under 800 miles it is 46·66 per cent.; under 1252 miles it is 75·00 per cent. Or the marches being computed by days, the ratio of attacks to marches per cent. stands thus: under 20 days = 8·029 per cent.; under 40 days = 17·762; under 60 days = 20·232; under 80 days = 40·540; under 100 days = 44·446; under 120 days = 50·000; over 120 days =50·000.

The extraordinary predisposition which marching induces is marked by the fact, that at the end of the third month the attacks of cholera to all the marches performed by the Madras native infantry were 61 per cent.

It has been generally supposed that cavalry suffer less on the march than infantry, from their less exposure and fatigue. This is probably correct; although Dr. Lorimer's tables make the proportion rather less than is commonly given.

According to this Report, for the whole number of marches, the respective corps of the Madras native service, give the following ratio of attacks, to marches, per cent. Among the infantry, 24·688 per cent.; among the cavalry, 20·270 per cent.; among the artillery, 50·000 per cent.; and among the sappers and miners, only 11·764 per cent. The returns from the artillery and the sappers and miners have only been available for the last few years. Dr. Lorimer, to account for the singular exemption of the sappers, mentions that they are, from the nature of their service, inured to the weather, being frequently under canvass, and also being men of low caste, they are not so restricted in their food.

The analogy between cholera, fever, and bowel complaints has led to the belief that these diseases prevail together, and certainly in many cases remittent fevers and dysentery prevail subsequent to an epidemic attack of cholera. Dr. Lorimer thinks, however, there is no uniform connexion between cholera, fever, and bowel complaints. His tables are certainly very full on this point, and seem satisfactory. It may not be uninteresting to give the whole sickness among the Madras native army since 1821. The aggregate number of men during this period has been 1,655,236; of cholera there have been admitted 22,347, and have died 8836 cases; of fevers there have been admitted 420,201, and have died 5946 cases; of dysentery and diarrhea, there have been admitted 67,564, and have died

4098 cases; of all other diseases there have been admitted 548,796, and have died 11,258 cases.

The ratio of deaths to strength per 1000 per annum, drawn from an average of the same period, are of cholera 5·3382; of fevers 3·5922; of dysentery and diarrhea 2·4751; of all other diseases 6·8014. The average deaths per 1000 per annum are, therefore, 18·2069, which is considerably below the mortality among the European troops, if cholera be included in the calculation.

At page 36 of this Report is a table showing the comparative admissions and deaths from cholera among the Madras native troops on the line of march and in cantonments. The ratio of admissions to strength per 1000 are for the line of march 20·9230, and for cantonments 8·5068; the ratio of deaths to strength per 1000 are for the line of march 8·6363, and for cantonments 3·2700; the ratio of deaths to admissions per 1000 are for the line of march 412·7698, and for cantonments 384·4074.

Dr. Lorimer's return evidently points out the three grand rules which ought to be attended to in all marches in India, viz. to shorten the marches as much as possible; to make them in as small bodies as possible; and, whenever it is practicable, to choose the dry months for the time of moving.

Although neither the conditions of the soil or of the atmosphere, or the state of the individuals who happen to be exposed to it, have anything more to do with cholera than as auxiliaries to its development or its reception, it cannot be doubted that the study of these phenomena, as it is the easiest, so also it is the most important part of the investigation of cholera.

In this direction only can we at present look for any practical results, and we receive Dr. Lorimer's able Report with great satisfaction as the first of a series which will place these matters on a certain basis. We think, however, that future reports should be more minute than this could possibly have been, as the returns which furnish it being retrospective are necessarily incomplete. We think if an extensive series of investigations were set on foot over the whole of India, inquiring minutely into all the attending circumstances of each attack of cholera, the results would in very few years be most important. The conditions of existence of the virus, and the predisposing circumstances being known, the prophylactic measures might be able to arrest its progress, and to limit its ravages. To accomplish this, would be a triumph worthy of that great Company, whose subjects and whose servants are alike the victims of this fatal and incurable disease.

The increased prevalence of cholera in India, since 1841, and its recent advance into Persia, has naturally given rise to the opinion that this disease may be expected again to travel in its old footsteps towards the colder countries of the northern and the western world. And there seem good grounds for this opinion. But if such an occurrence should take place, it is possible that at the expense of present suffering, great benefit would result to the whole human race. We would fain hope that if cholera should again traverse Europe, the laws of its diffusion might be more surely indicated, its true nature better illustrated, and a more successful treatment discovered.

Admitting this possible invasion to be probable, it would be the part of the

European governments to endeavour to institute so minute an inquiry into the conditions of the media favoring the spread of the disease, that nothing should be left unnoticed. This can only be done by combined effort working in one direction; it ought not to be left to individual effort, which is partial, limited, and therefore comparatively unproductive. From the moment cholera appeared in Europe, government should combine all classes of observers, and call in the aid of every science. The chemists and the meteorologists, the geologists and the physicians, should all be called upon to supply their observations, independent indeed, but all essential. Facts of all kinds should be noted without regard to opinion, and till the close of the epidemic no premature generalization should be attempted.

Some rules, simple indeed, yet not unimportant, may be deduced from our present knowledge, as applicable in the event of another invasion of the disease.

1. As there is no valid evidence of their use, quarantines, cordons, and other general measures of seclusion may be properly abandoned.

2. At the same time, all unnecessary exposure of patients, their excretions, clothing, &c., in situations frequented by other persons, and all direct intercourse between the sick and the well, not necessary for the comfort, nursing, and proper medical treatment of the patients, should be avoided as a measure of wise precaution, and on the ground of possible evil. But no valid evidence exists for justifying the neglect of the sick, in any the least degree, by their relatives or attendants, on the ground of personal danger: we do not contemplate the possibility of neglect by the medical profession, under any considerations whatsoever, and therefore do not name this.

3. As dampness and vegetable and animal effluvia seem powerful agents in spreading the virus, the dwellings of the poor should not only be made as dry and airy as possible by draining, ventilation, and whitewashing, &c., but government ought to take into consideration the site of dwelling-houses, and should compel the builder not only to follow certain rules in the construction of his tenements, as is done now by the Building Act, but should also compel him to choose a proper and dry locality, or to take means to render it so.

4. The cholera hospitals should be removed from the low and damp parts of the town where the disease chiefly prevails, and should be on dry soil, as high as possible, and built with even a freer circulation of air than other hospitals.

5. In some places, considering the peculiar way cholera is limited by a street or road, apparently from some attractive force exerted towards it by certain soils or other local influence, it might be advisable to effect, if such a thing were possible, the removal of the inhabitants from a part of the town severely affected with the complaint.

6. The profession should endeavour to discover some certain method of diagnosis, by which the true epidemic might be distinguished from those pseudo-choleraic cases which accompany it, and which lead to wrong notions of the nature of the disease, and erroneous methods of treatment.

7. All classes should guard against the predisposition which seems essential to the action of the agent. This is increased by disorders of the digestive organs, particularly by watery diarrhea; and by all means physical or mental which lower the strength and diminish the tone of the system. For instance, the apprehension of the disease is known to predispose to it in a very singular way. Dr. Lorimer mentions a native regiment which has been fortunate enough to have escaped cholera up to the present date. The commanding officer attributes this to the custom prevalent in the corps of offering up sacrifices previous to a march; the confidence the men have that cholera will afterwards not attack them seems certainly to have been a protective influence. The increased number of attacks of cholera which take place at night seem to show that at this time the system is peculiarly liable to the action of the agent.

Our space admonishes us that we must take leave of our subject and the authors from whose pages we have drawn much and valuable instruction. Although we differ considerably from Dr. Kennedy, we have derived great pleasure from a perusal of his work, which is evidently the composition of an able physician and a clever man. Of Dr. Lorimer's excellent Report we have already spoken; and Dr. Copland's great and deserved reputation is sufficient to warrant that his article on Cholera, is written with his usual tact and ability, although we have not hesitated to dissent from the confident opinion he has formed of the contagion of the disease. We think that, on this point, he is not merely wrong, but has allowed his prepossessions to blind him to obvious truths, and even to disregard palpable facts.

---

